# Improving transitional care communication for older Australians from hospital to home: Co‐design of the TRANSITION tool

**DOI:** 10.1111/hsc.13816

**Published:** 2022-05-04

**Authors:** Jacqueline Allen, Alison M. Hutchinson, Rhonda Brown, Patricia M. Livingston

**Affiliations:** ^1^ 2541 School of Nursing and Midwifery Monash University Clayton Vic. Australia; ^2^ School of Nursing and Midwifery Centre for Quality and Patient Safety Research Institute for Health Transformation Deakin University Geelong Vic. Australia; ^3^ School of Nursing and Midwifery Deakin University Geelong Vic. Australia

**Keywords:** co‐design, communication, older adults, qualitative methods, transitional care

## Abstract

This study aimed to develop and evaluate a communication tool to guide transitional care for older patients. Using experience‐based co‐design, a communication tool resulted from the triangulation of data collected from three study phases. From 2015 to 2016, semi‐structured interviews and co‐design focus groups were undertaken with older patients, carers and healthcare practitioners across acute, rehabilitation and community settings. The evaluation phase, conducted in 2017–2018, involved use of the communication tool by healthcare practitioners in a multidisciplinary care team with older patients in acute care and semi‐structured interviews with healthcare practitioners about the acceptability and feasibility of the tool. A total of 103 patients, carers and healthcare practitioners took part. In semi‐structured interviews, patients and carers reported needing to become independent in care transitions, which was supported by discussing the transitional care plan with healthcare practitioners. Interviews with healthcare practitioners identified that their need for fast and safe care transitions was supported by team discussion and by engaging patients and carers in their transitional care plan. Co‐design focus group participants identified principles guiding transitional care including patient‐centred communication. Data collected from semi‐structured interviews and co‐design focus groups were used to develop a prototype communication tool to guide conversations about discharge care between healthcare practitioners and older patients. Following use, healthcare practitioners reported that the communication tool was feasible and acceptable although some nurses perceived that transitional care was not their role. The communication tool provides an evidence‐based resource for ward nurses to support transitional care continuity in multidisciplinary models.

**What is known about this topic?**

- Optimal communication is challenging during care transitions because of rapid bed turnover.
- Few communication tools emphasising discussion between practitioners and older patients in transitional care have been developed.
- Experience‐based co‐design has not been used in improving transitional care communication for older patients.

**What this paper adds?**

- The TRANSITION tool is for use by ward‐based nurses to guide continuing conversations with patients, monitoring and referral to allied health professionals prior to the patient's discharge.
- Further research is required to evaluate the effectiveness of the TRANSITION tool across the patient transition into the community.
- The role of patients and carers in using the TRANSITION tool to take part in their own care transitions requires further investigation.

## 1 INTRODUCTION

Quality communication between healthcare practitioners and older adults living with chronic disease during care transitions from hospital to home is challenging. This is partly because of system‐based barriers limiting available time for communication including rapid bed turnover and service fragmentation (Goncalves‐Bradely et al., 2016). Suboptimal transitional care and ineffective communication contribute to re‐admission, medication misadventure and falls (Goncalves‐Bradely et al., 2016). Using experience‐based co‐design (co‐design), this qualitative study aimed to develop a communication tool to guide conversations about transitional care needs between healthcare practitioners and older patients. In this paper, we report on the co‐design methods we used to triangulate three study phases resulting in the development of the communication tool. Detailed discussion of each individual study phase has been published elsewhere (Allen et al., 2018, 2020).

### 1.1 Transitional care and communication

Transitional care refers to interventions that promote safe and timely transfer of patients between levels of care and across care settings (Coleman et al., 2006; Naylor et al., 2013). Previous research has demonstrated the effectiveness of advanced practice nurses, self‐management coaching, discharge case management and multidisciplinary models in facilitating care transition and preventing re‐admission (Coleman et al., 2006; Hickman et al., 2015; Jeffs et al., 2017; Naylor et al., 2013). However, effective communication to improve care transition is a persistent challenge in practice.

Recent research has identified the need for more person and family‐centred transitional care emphasising improved communication between healthcare practitioners and older adults (Aase & Waring, 2020). However, in transitional care research, communication has been largely understood as the exchange of information between healthcare practitioners (Goncalves‐Bradely et al., 2016; Jeffs et al., 2017). A systematic review by Foster and Manser (2012) found that patient hand‐over communication at transitions between providers focussed on information exchange in the form of patient checklists. However, there is mixed support for the effectiveness of checklists and a need to examine other forms of communication such as mnemonics to guide hand‐over communication (Foster & Manser, 2012). A study by Jeffs et al. (2017) employing an expert panel to identify effective nurse‐led transitional care, also recommended further research to examine communication in the form of discussion and conversation between healthcare practitioners, older patients and informal unpaid carers (carers). In a recent meta‐synthesis study conducted in the early phases of the current study, we identified questioning, discussion, information provision and information seeking as effective communication strategies for healthcare practitioners, older patients and their carers in navigating care transitions (Allen et al., 2017). Despite this research, few studies have developed a communication tool emphasising questioning and discussion between practitioners and older patients in transitional care. A tool promoting communication between practitioners and older patients could improve person‐centred transitional care. This study aimed to co‐design a communication tool to guide conversations about transitional care needs between healthcare practitioners and older patients returning to the community.

## 2 METHODS

The consolidated criteria for reporting qualitative studies (COREQ) were used to guide the reporting of the methods (see File S1). The study was informed by co‐design principles (Bate & Robert, 2007) and data collection tools and interview guides were developed by our earlier meta‐synthesis review (Allen et al., 2017). The lead author is a community nurse with a research background in improving discharge and transitional care of older patients from hospital to home. Any potential influence of the lead author's preconceptions was interrogated by co‐authors during checks for dependability and confirmability as part of data analysis in each phase.

### 2.1 Conceptual framework

The conceptual framework is social constructivism, understood as social processes simultaneously creative of and created by people through interactions with their social context, and with each other (Silverman, 2013). Social constructivism was selected because it conceptualizes interactive social processes, including communication, that are the focus of the study aim. User experience was the principal form of knowledge driving the study and refers to how the user of a health service feels and thinks about the service while they are using it (Bate & Robert, 2007). The social world, particular to the healthcare environment, shapes users’ interpretations of the meaning of their experiences of healthcare, and users’ interpretations of the meaning of their experiences of healthcare can in turn shape the social world of the relevant healthcare environment. Hence, user experience in healthcare contexts, including transitional care, is itself a social process and formed the principal meaning unit for this qualitative investigation.

### 2.2 Design

Co‐design was used with mixed methods data collection across three phases. Co‐design is a participative action research method where service users, including patients, carers/families and healthcare practitioners and providers collaborate to design health services (Bate & Robert, 2007). In a survey study, Donetto et al. (2014) found that co‐design has been used in several western countries in a range of specialisations including cancer, mental health, drug and alcohol and emergency departments. To date, few studies have employed co‐design to involve service users in optimal transitional care communication and experiences.

In this paper, we report on three study phases from which the results were triangulated to develop the communication tool. The first phase was a context inquiry, conducted from 2015–2016, including semi‐structured interviews with older patients and their carers, and with healthcare practitioners to ascertain the practice context. The second phase was undertaken in 2016 and included co‐design focus groups where participants were invited to provide feedback on the findings from the context inquiry, derive principles from which to design the communication tool, and check the appropriateness of the prototype communication tool. In the final phase, conducted from 2017 to2018, healthcare practitioners used the tool with an older patient and then participated in a semi‐structured interview regarding the acceptability and feasibility of the tool. Drawing on Teddlie and Tashakkori (2009) and Ritchie and Ormston (2014) we understand acceptability and feasibility as the suitability and practicality of the tool including enabling and constraining factors. Data from the final phase informed the modification of the communication tool.

### 2.3 Ethics approvals

Ethics approvals for the context inquiry, co‐design and evaluation phases of the study were obtained from participating healthcare organisations and from the University. Participation in each phase was voluntary. Participants signed a consent form following explanation of the study and requirements guided by an Explanatory Statement as relevant to the study phase. All data were de‐identified. All interviews were audio‐recorded for transcribing.

### 2.4 Context inquiry

#### 2.4.1 Setting

A large metropolitan public healthcare network, general practice and two community care organisations in the Australian city of Melbourne formed the setting for the context inquiry. Patients and carers, and healthcare practitioners were recruited from two inpatient sites including a ward providing acute general medicine services, and a ward providing rehabilitation services for older people. Healthcare practitioners were recruited from aged care and rehabilitation services at the public healthcare network, general practice and from the participating community‐based organisations.

#### 2.4.2 Participants: patients and carers

Using purposive sampling for maximum variation regarding age and country of birth, up to 20 patients, plus or minus their carer, were selected to participate in a semi‐structured interview. Patients were eligible when they had transitioned from hospital to home, were aged 70 years or older, lived with at least one chronic health problem, and spoke English sufficiently to provide informed consent. Carers were unpaid carers providing support to the older patient and included family and friends. Carers were nominated by participating patients and were eligible to participate when they were at least 18 years of age and spoke English sufficiently to provide informed consent. Patients and carers with cognitive impairment were excluded.

Codes and categories that were similar to each other were identified following 12 interviews. Because of the difference between acute and rehabilitation ward settings, we conducted an additional eight interviews to check for new codes and categories. No new codes and categories emerged indicating that data saturation was established with a total of 20 interviews.

#### 2.4.3 Participants: healthcare practitioners

To be included, healthcare practitioners needed to be employed by a participating organisation and provide transitional care to older patients and their carers. Purposive sampling, using maximum variation for healthcare discipline and setting, was used to invite participants for a semi‐structured interview and included multidisciplinary practitioners across acute, rehabilitation and community settings.

During data collection that commenced in the acute general medicine inpatient ward, participants reflected on the range of publicly funded programs used by older adults undergoing care transitions and living with multiple chronic diseases in the acute, rehabilitation and community settings. These programs included general medicine (acute inpatient), Hospital in the Home (acute community), Geriatric Evaluation and Management (rehabilitation inpatient), Aged Care Consultancy and Triage (rehabilitation inpatient), Post‐acute Care (rehabilitation community), Transition Care Program (rehabilitation inpatient and community), Aged Care Case Management (community), District Nursing (community) and General Practice (community/primary care). To describe the context of care transitions for older adults, we aimed to include at least two healthcare practitioners per program and at least one healthcare practitioner per discipline as relevant to the program. We identified similar codes and categories following 30 interviews. To ensure data saturation within each of the nine health programs we conducted an additional 17 interviews with no new codes or categories emerging. Following completion of 47 interviews, we concluded that we had reached data saturation.

#### 2.4.4 Data collection tools and interview guides

A semi‐structured interview guide was developed using the recommendations of Bate and Robert (2007) and using the meta‐synthesis review conducted earlier (Allen et al., 2017). All participants completed a demographic questionnaire including information about the participant's: age; gender; country of birth; health status (for patients and carers); current role and healthcare discipline (for healthcare practitioners). The semi‐structured interview guide for patients and carers (See File S2) prompted responses regarding experiences of transitional care including what was valued and what was missed. The semi‐structured interview guide for healthcare practitioners (See File S2) prompted responses about experiences of transitional care provision including facilitators and barriers.

#### 2.4.5 Procedure

Senior nurses on each ward were invited to identify patients who met the inclusion criteria and introduce the study to them. With patients’ permission and using the ethics approved Participant Information and Consent Form (PICF), the first author explained participation in the study and then conducted a face‐to‐face semi‐structured interview with the patient in their own home at least one week following hospital discharge. The first author is female and is an experienced registered nurse, educationally prepared at Masters level with skills in interviewing patients and research participants. Patients who agreed nominated their carer who was also invited to participate in the interview.

Management at each site nominated key personnel involved in transitional care who met the inclusion criteria. With permission and using the PICF for healthcare practitioners, the first author invited practitioners to participate in the study and conducted face‐to‐face semi‐structured interviews in participants’ place of employment during a scheduled meal break or in an office at the university. Permission was requested from patients who had participated in the semi‐structured interviews for the first author to contact their general practitioner (family physician). The first author invited general practitioners to participate in the study and sent the PICF for healthcare practitioners by email. The first author then contacted the general practitioner by telephone, explained participation in the study and conducted a telephone interview at an agreed time.

### 2.5 Co‐design focus groups

#### 2.5.1 Setting

The setting was the same as noted earlier.

#### 2.5.2 Participants

In accordance with co‐design principles (Bate & Robert, 2007), patients, carers and healthcare practitioners were included together as participants in the co‐design focus groups. Each focus group comprising the same participants met on three separate occasions. Participants were recruited purposively, and inclusion criteria were the same as reported above for the context inquiry.

All patients and carers, and healthcare practitioners invited to participate in the context inquiry were invited to participate in the co‐design focus groups. One patient, one carer and one healthcare practitioner responded. The remaining patients declined to participate due to frail health. Remaining carers declined to participate due to lack of time. Healthcare practitioners declined further participation because of lack of time. Therefore, a recruitment invitation was included in the staff newsletter for the healthcare network and each community organisation was sent a recruitment invitation. The recruitment strategy resulted in an additional four healthcare practitioners. One of the healthcare practitioners was also an informal carer. In accordance with Silverman (2013), seven participants were considered an adequate number for interaction within a small group. Three co‐design focus groups were conducted.

#### 2.5.3 Data collection tools and interview guides

For the context inquiry study, data collection tools and interview guides were developed from the meta‐synthesis review conducted earlier (Allen et al., 2017). In the first co‐design focus group, all participants completed a demographic questionnaire including information about their: age; gender; country of birth; health status (for patients and carers); education, current role and healthcare discipline (for healthcare practitioners). In focus groups 1 and 2, we presented a summary of the themes from the findings in the context inquiry. In focus group 1, the questions ‘Do the findings make sense? How/how not?’ were used to guide the discussion. In focus group 2, participants were invited to imagine the ideal discharge and care transition. We used the question ‘What are the “must haves” in discharge and transitional care?’. In focus group 3, we presented the prototype communication tool. We used the questions ‘Does the tool make sense?’ and ‘What could be improved?’.

#### 2.5.4 Procedure

The focus groups were scheduled at three time points with a one‐week interval between the first and second focus groups, and a 2‐week interval between the second and third focus groups to allow sufficient time to develop the communication tool. The three focus groups were facilitated by the first author using the interview guides noted above. A research assistant supported each of the focus groups by assisting participants to complete the demographic questionnaire and by observing the group dynamics and documenting these in field notes.

### 2.6 Evaluation

#### 2.6.1 Setting

The setting comprised two acute medical wards in one public health network in suburban Melbourne Australia. This public health network was a different organisation from the organisation that participated in the context inquiry and co‐design focus groups. To maximise sampling heterogeneity, healthcare practitioners were recruited from a range of disciplines including nursing and allied health.

#### 2.6.2 Participants

Purposive sampling was used to recruit healthcare practitioners from participating wards and programs who were aged 18 years or older and conducted transitional care of older patients. Nurse Unit Managers at the participating wards and management staff at the health network invited healthcare practitioners who met the inclusion criteria to consider participation in the study. With healthcare practitioners’ permission and using the ethics approved PICF, the lead author or research assistant explained all requirements of participation in the study to them including details about the communication tool, use of the tool and evaluation steps. Codes and categories that were similar to each other were identified following 18 interviews. Five additional interviews were undertaken with no new codes and categories emerging. We, therefore, considered that we had reached data saturation after 22 interviews.

#### 2.6.3 Data collection tools and interview guides

Data collection tools and interview guides comprised screening and demographic questionnaires and a semi‐structured interview guide. These were developed from previous study phases. Screening and demographic questionnaires included information about the participant's age and gender. The interview guide (See File S2) included questions about the healthcare practitioner's perception of the acceptability and feasibility of the tool and suggested improvements to the tool.

#### 2.6.4 Procedure

During recruitment, healthcare practitioner participants were given education about how to use the communication tool one‐on‐one by the first author or by the research assistant. In these education sessions, practitioners were asked to engage at least one patient or carer on the ward in a conversation about their care transition using the questions in the prototype communication tool as a guide. Practitioners were given a copy of the semi‐structured interview guidelines to use as a guide in making notes regarding their experience. Following use of the tool with at least one patient or carer, practitioners were invited to participate in a semi‐structured interview. Interviews were conducted by either the first author or a research assistant at the participant's work location during a scheduled meal break. The research assistant was a female registered nurse educationally prepared at Bachelor degree level. The research assistant in the evaluation study phase was different from the research assistant who assisted with the second study phase.

### 2.7 Data analysis

Quantitative data were entered into SPSS for descriptive statistics including frequencies and means. Qualitative data were thematically analysed by the lead author in collaboration with the other authors. A summary of each study phase, data collection tool and interview guidelines, data and data analysis is presented in Table 1.

**TABLE 1:** Summary of data collection tool and interview guidelines, data and data analysis for the three study phases

Study phase	Data collection tool and interview guidelines	Data^a^	Data analysis
*Context inquiry*			
Semi‐structured interviews (patients and informal carers)	Demographic questionnaire Interview guide Patient and informal carer experience of transitional care	Demographic questionnaires Interview audio‐recordings and transcripts	Frequency and descriptive statistics (demographic questionnaires) Thematic analysis (semi‐structured interview transcripts)
Semi‐structured interviews (healthcare practitioners)	Demographic questionnaire Interview guide Healthcare practitioner experience of providing transitional care	Demographic questionnaires Interview audio‐recordings and transcripts	Frequency and descriptive statistics (demographic questionnaires) Thematic analysis (semi‐structured interview transcripts)
*Co‐design focus groups*			
Three sequential focus groups (healthcare practitioners, patient, carers)	Demographic questionnaire Interview guides Consideration of findings from context inquiry (co‐design focus groups 1 and 2) Principles to guide the development of the tool (co‐design focus group 2) Feedback re the prototype tool (co‐design focus group 3)	Demographic questionnaires Focus group interview audio‐recordings and transcripts	Frequency and descriptive statistics (demographic questionnaires) Thematic analysis (focus group interview transcripts)
*Evaluation*			
Semi‐structured interviews (healthcare practitioners)	Demographic questionnaire Interview guide Healthcare practitioner experience of using the TRANSITION tool	Demographic questionnaires Interview audio‐recordings and transcripts	Frequency and descriptive statistics (demographic questionnaires) Thematic analysis (semi‐structured interview transcripts)

^a^ No participant had a pre‐existing relationship with any member of the research team.

Interviews were thematically analysed separately for each study phase. Thematic analysis was an iterative process and supported by the Framework Approach (Spencer et al., 2014). Using the research aim and rationale for data collection as guides, thematic analysis involved comparing and contrasting of codes and categories within and where relevant between interviews to derive a plausible formulation of the data. All authors coded raw data files and interrogated interpretations made by the first author.

### 2.8 Triangulation of findings and rigour

Triangulation between study phases was employed to co‐design the communication tool in two parts; (1) tool development; and (2) evaluation. Findings from the context inquiry were presented to participants in the co‐design focus groups for validation, for their consideration of principles to guide the development of the tool and to provide feedback on the prototype tool. Findings from the evaluation were used to refine the tool. Triangulation of findings was further supported during meetings where all authors interrogated interpretations made by the first author to develop, evaluate, and refine the communication tool.

We applied the principles of trustworthiness as proposed by Lincoln and Guba (1985) to describe the process of triangulation: credibility, transferability, dependability and confirmability. Credibility is supported through prolonged observation occurring over the three study phases (Silverman, 2013; Teddlie & Tashakkori, 2009). Because multiple data sources were used, a multi‐perspective description of communication in transitional care practice between healthcare practitioners, older patients and carers also supports the credibility of the study (Silverman, 2013). Detailed description of the study context was the focus of the context inquiry, which should enable others to consider how the communication tool, or elements of it, might be applied to similar contexts of care.

Data analysis for each phase of the study and related inferences about the data including the triangulation of findings, were examined for consistency through dependability and confirmability checks (Silverman, 2013; Teddlie & Tashakkori, 2009). In these checks, all authors reviewed raw data files and interrogated interpretations made by the first author. Confirmability was further supported through the co‐design focus groups, when participants had the opportunity to provide feedback on the findings from the context inquiry and on the communication tool. In triangulating the findings, the first author referred to field notes she made throughout data collection and to field notes made by the research assistants, which also supported dependability and confirmability.

## 3 FINDINGS

A total of 103 patients, carers and healthcare practitioners took part across the three study phases. Table 2 presents participant characteristics, information about data saturation and details regarding interviews for the context inquiry, co‐design focus groups and evaluation phases. A summary of the main themes from each study phase including example quotes is presented in Table 3.

**TABLE 2:** Participant characteristics, data saturation and interview details: context inquiry, co‐design focus groups and evaluation study phases

Rationale for data collection	Participants	Data saturation	Duration and place of interviews	Interviewer details^a^
*Context inquiry: patients and carers*				
To describe older patients’ and informal carers’ experience of transitional care	20 semi‐structured interviews, 26 participants: 13 interviews with individual patients 6 interviews with patient and carer dyads 1 interview with individual carer Demographic details for all 19 patients: Mean age 82.6 years (*SD* 6.6 years, range 72–94 years) 11 were female Demographic details for all 7 carers: Mean age 68.9 years (*SD* 14.1 years, range 45 to 88 years) 5 were female 6 family members (4 spouses, 1 daughter, 1 son) 1 friend and neighbour	Similar codes and categories were identified following 12 interviews. An additional eight interviews were undertaken with similar results indicating that data saturation was established.	Interviews were on average 37 min duration Interviews were conducted in patients’ homes	First author: Female Experienced registered nurse with skills in patient/practitioner interviewing Educationally prepared at Master of Psychology level
*Context inquiry: healthcare practitioners*				
To describe healthcare practitioners’ experience of providing transitional care to older patients across acute, sub‐acute and community‐based settings and programs	47 semi‐structured interviews, 48 participants 47 interviews with individual practitioners 1 interview with 2 registered nurses (acute) (due to their need to continue to staff the telephones) Demographic details: Mean age 44 years (*SD* 11.6 years, range 23–64 years) 40 were female 25 registered nurses (acute, rehabilitation, community) 2 enrolled nurses (acute, community) 8 social workers (acute, rehabilitation, community) 7 medical practitioners (acute, rehabilitation, community) 2 physiotherapists (acute, community) 2 occupational therapists (acute, rehabilitation) 1 pharmacist (acute) 1 case manager (community)	Similar codes and categories were identified following 30 interviews. An additional 17 interviews were undertaken with similar results supporting data saturation.	Interviews were on average 26 min duration Interviews were conducted at: Participants’ place of employment (43 interviews) University (3 interviews) By telephone (1 interview)	First author (details as above)
*Co‐design focus groups*				
Focus group 1: To confirm findings from semi‐structured interviews undertaken in the context inquiry Focus group 2: To consider principles to guide the development of the communication tool Focus group 3: To discuss the appropriateness and acceptability of the prototype communication tool	Three sequential focus groups, the same 7 participants were invited to each focus group 1 ex‐patient 1 informal carer 1 health provider and informal carer (community) 1 social worker (sub‐acute care) 3 registered nurses (acute, sub‐acute, community) Participant attendance at co‐design focus group 1: All 7 participants Participant attendance at co‐design focus group 2: 3 participants (1 ex‐patient, 1 informal carer, 1 health provider and informal carer) Participant attendance at co‐design focus group 3: 5 participants (3 registered nurses (acute, sub‐acute, community), 1 social worker (sub‐acute care), 1 health provider and informal carer) Demographic details: Mean age 52 years (*SD* 14.5 years, range 27–76 years) 6 were female	Not relevant given rationale for data collection	Each focus group was of 60 min duration All 3 focus group interviews were conducted at: A meeting room at the University	First author (details above) Supported by research assistant
*Evaluation*				
To evaluate the prototype communication tool for feasibility and acceptability from healthcare practitioners’ perspectives	22 semi‐structured interviews, 22 healthcare practitioners, demographic details: 12 aged 30–49 years 17 were female 17 nurses 2 physiotherapists 1 occupational therapist 1 social worker 1 pharmacist	Similar codes and categories were identified following 18 interviews An additional 5 interviews were undertaken with similar results indicating that data saturation was established.	Interviews were on average 14 min duration Interviews were conducted in participants’ place of employment	First author (details as above) Research assistant Experienced registered nurse with skills in patient/practitioner interviewing Educationally prepared at Bachelor level

^a^ Interviewers did not have a relationship with any study participants. Participants knew that the interviewers were registered nurses.

**TABLE 3:** Main themes and example quotes: context inquiry, co‐design focus groups, evaluation

Data	Main themes	Example quotes
*Context inquiry*		
Interview transcripts older patients and carers	Four main themes reflecting social and communication processes were identified	
	Needing to become independent	‘Coming home. To be independent again that I can live my life the way I want to and if I want an extra snooze I’ll go. It's the independence.’ Ex‐patient Participant 24
	Caring relationships with healthcare practitioners	‘We for the first time ever a nurse came out and she brought us to the car. And even my friend, she couldn't believe it.’ Ex‐patient Participant 13
	Discussing and negotiating the transitional care plan	‘Well they said I was going home, going home all day. And then it got to when the blood didn't get in there. Then they said oh it'll only take three hours and you'll be able to go. Gwen [friend] got on to it and she said she's definitely not going to pick me up to take me home to an empty house in the dark at that time of night after I’ve just had the blood.’ Ex‐patient Participant 25
	Learning to self‐care	‘I knew about attaching the night bag and all of that from watching them [the ward nurses]. So one of the district nurses changed it a couple of times and then I said, ‘Look, you can spend your time better elsewhere, I’m quite capable of doing it.’ Ex‐patient Participant 16
Interview transcripts healthcare practitioners	Three main themes reflecting social and communication processes were identified	
	Rapid and safe care transition	‘We've got the push to get people out.’ Medical practitioner, acute ward, Participant 10
	Discussing as a team	‘A lot of the time from a medical point of view this person is healthy enough to go home. And then they will say, we're not sure if their home set up is practical or if they are going to be able to walk in and out of the house. So that's when allied health might get involved. And then sometimes we're explaining to the medical team our perspective and we also like to hear what is going on for the patient medically as well to guide our recommendations.’ Physiotherapist, sub‐acute, Participant 15
	Engaging patients and carers	‘We definitely do get the patient involved as well to try and encourage them back to their premorbid stage. You try to get them back home.’ Registered nurse, acute ward Participant 2
*Co‐design focus groups*		
Focus group interview transcripts	Focus group participants endorsed the findings from the context inquiry. Four themes were identified.	
	Needing bed access	‘Everything we do within the acute environment now is focussed on trying to meet those targets, get patient flowthrough.’ Registered nurse, acute Participant 2
	Navigating discharge and care transition	‘You have to be really proactive, we were ringing the ward, the nurse or the people that were looking after mum were sort of saying so what's happening with the discharge? She was going from here to rehab, so that was a bit more straightforward’ Informal carer Participant 4
	Proactive communication	‘It's really important to build into [discharge care], is communication with the families. And I know how difficult that can be.’ Informal carer, Participant 5
	Supporting and educating the person and carer	‘Sometimes the carers themselves need support from the people who are making the decisions to understand.’ Informal carer, Participant 5
*Evaluation*		
Interview transcripts healthcare practitioners	Four main themes were identified:	
	The tool is feasible and acceptable	‘When it comes to discharge, I think it [the TRANSITION tool] covers all the points on discharging someone from the hospital. All the questions apply. … And finding out how they feel and what the future will be like for them.’ Participant 7, ward nurse
	Knowing what to ask	‘Being able to ask those questions that I do not necessarily ask. In terms of how well they are looking after themselves at home. Are they able to cook and clean for themselves? Because we don't always ask our patients that as nurses.’ Participant 1, ward nurse
	Finding more information	Interviewer: ‘What did you find most valuable on the tool?’ Participant 16, ward nurse: ‘I just found knowing more about the injury risk at home. We find that so many patients are coming back from hospital, they are home for a few weeks and then they are back in hospital because of something else. Probably more risks at home.’
	Transitional care is not my role	‘From a nursing perspective, I guess I don't always have time or don't see my need to have to dig too deep into some of these questions [the TRANSITION tool] for the patient.’ Participant 15, ward nurse

### 3.1 Context inquiry

#### 3.1.1 Patients and carers

A total of 20 interviews were conducted with 26 patients and carers. Thirteen patients were interviewed alone, six patients were interviewed together with their carers (6 carers) and one carer was interviewed alone. All patients and carers emphasised that becoming independent was the problem to be solved in care transitions. They reported that caring relationships with healthcare practitioners and discussing and negotiating the transitional care plan supported their return to independence after hospital discharge.

Patient and carer participants described caring relationships with healthcare practitioners in terms of feeling cared for as a person, feeling included and respected. Participants noted that they or their informal carers discussed and negotiated their transitional care plan with healthcare practitioners. According to patient participant 25, ‘Well they said I was going home, going home all day. And then it got to when the blood didn't get in there. Then they said oh it'll only take three hours and you'll be able to go. Gwen [friend] got on to it and she said she's definitely not going to pick me up to take me home to an empty house in the dark at that time of night after I’ve just had the blood.’ This quote is one example of how the informal carer disagreed with healthcare practitioners regarding the discharge plan and arranged for discharge to be delayed to the following morning to support the older patient's care following a blood transfusion.

#### 3.1.2 Healthcare practitioners

Interview findings with 48 healthcare practitioners from nursing, medicine and allied health across acute, rehabilitation and community settings identified that healthcare practitioners perceived fast and safe care transitions as the problem that they needed to solve. Participants reported that they used communication processes including discussing as a team and engaging patients and carers in their transitional care plan. They reflected that team discussions were important for raising concerns and also for considering the different perspectives from nursing, allied health and medicine to optimise the transitional care plan. Participants further explained that they engaged patients and carers to focus on their goals and expectations, include family and support the patient towards independence. According to a registered nurse participant in acute care (participant 2), ‘We definitely do get the patient involved as well to try and encourage them back to their premorbid stage. You try to get them back home.’ Some healthcare practitioners reported difficulties engaging and motivating patients and carers in transitional care because older adults and carers could experience cognitive difficulties, lack of interest in their own care and lack of insight into their care needs.

### 3.2 Co‐design focus groups

Of the seven participants recruited, all took part in the first co‐design focus group, three participants took part in the second focus group and five participants took part in the third focus group. Participants who were unable to attend each focus group noted that this was due to insufficient time or illness. In the first and second co‐design focus groups, participants endorsed findings presented to them from the context inquiry. The health practitioner participants also reported that the problem to be solved in transitional care was ‘needing the bed’. They noted that navigating the care transition, proactive communication and support for the person and carer assisted during transitional care. Patient and carer participants emphasised their need to be acknowledged and to be included in discharge communication. According to an informal carer (participant 5), ‘It's really important to build into [discharge care], communication with the families. And I know how difficult that can be.’ Participants in the first and second co‐design focus groups identified person‐centred principles to support communication (see Table 4). These principles were used to guide the development of questions comprising the prototype communication tool. In the third co‐design focus group, participants endorsed the prototype communication tool and suggested additional prompts and questions resulting in the trial version of the communication tool, the TRANSITION tool (presented in Table 5).

**TABLE 4:** Principles identified from co‐design focus groups 1 and 2

Eight principles were identified for quality transitional care to guide the development of the communication tool.
Healthcare practitioners need to remain patient‐centred
Healthcare practitioners need to ensure patient flow through the healthcare system
Healthcare practitioners need to use multidisciplinary teams and aged care expertise regarding assessment, planning (cognition, mobility and falls risk, side effects of medications) and service navigation in the community
Healthcare practitioners, patients and informal carers need to relate respectfully, effectively and empathically with each other
Healthcare practitioners should involve the patient, family and carers in discussions about discharge and transitional care
Older patients need quality and safe medication care and preparation at discharge
Patients, carers and families need education and support, including emotional support, in regard to discharge and care transitions

**TABLE 5:** Communication tool: the TRANSITION tool

Trial version	
Domain	Questions and prompts
**T**ime: making time to ask questions and listen	Making time to ask questions and listen about being on the ward, being at home and preparing to leave the ward.
**R**elationships: getting to know the person and family/carer	Building caring relationships between the patients, carers and healthcare practitioners Does the person have family/carer? Can the carer provide care? Is it acceptable to discuss care with the carer?
**A**dmission to hospital: introduction to the ward and what to expect	Introduction to the ward and to healthcare practitioners–different doctors, allied health and nursing practitioners may provide care
***N*** (Kn)owing and memory: what is the person's memory like?	How does the person function at home/remember to do the usual things (making a hot drink, making a meal, eating, sleeping, taking medicines, conversation, getting out of bed, getting dressed appropriately for weather and activity)?
**S**ervices at home: Who visits already and who might need to visit?	Who normally visits at home to assist with care? Does the person live alone – they may need a little more assistance to fully recover? Do services need to visit more regularly in the immediate follow‐up period? Is there a need for formal services after discharge?
**I**njury risk at home: What is the home environment like?	How does the person move around at home? Do they fall? Hazards and risks (stairs, rugs, clutter creating tripping risks)? Do the person and carer know about safety alarms at home and how to access these?
**T**ablets and medicines	How does the person manage to take their medicines? Who helps them to take their medicines? Do they know about aids from their local chemist that can help such as blister packs?
**I**nstructions and self‐care education	What do the person and carer need to know about looking after themselves at home? What information do they need? Who do they need to ask about self‐care at home? The first week home is challenging and confidence is critical. Encouragement, positive attitude and acknowledging the small steps are important.
**O**rganisation of discharge: Where is the discharge plan up to and who knows about it?	Has the person and carer been involved in the discharge plan? Has the carer/family been notified of changes in the discharge plan? Where is the person going to go?
**N**eeds and concerns: note any other concerns	Any other concerns about discharge and going home? Any concerns about being at home?

Domain	Questions and prompts	Date completed
**Modified version**		
**T**ime – Make time to talk		
**R**elationship – Get to know the patient		
**A**dmission – Introduction to the ward and what to expect		
***N*** (Kn)owing and memory	What is the person's memory like? Functioning at home? Taking their medicines?	


**S**ervices at home	Who visits already and who might need to visit? Who assists at home? Need for formal services?	


**I**njury risk at home	What is the home environment like? Hazards and risks at home (stairs, rugs)? Falls? Safety alarms at home?	


**T**ablets and medicines	How does the person manage their medicines? Taking their medicines? Are aids required – community pharmacist?	


**I**nstructions and self‐care education	What does the person and family need to know for them to look after themselves at home and be safe? Where to go with questions?	


**O**rganisation of discharge	At what stage of development is the plan? How is the patient returning home?	


**N**eeds and concerns	Are there any other concerns about being in hospital, about going home?	

### 3.3 Evaluation

#### 3.3.1 Healthcare practitioners

A total of 22 healthcare practitioners from nursing, occupational therapy, physiotherapy, social work and pharmacy took part in the evaluation. All six medical practitioners on each medical ward were invited to take part; however, they declined due to lack of time. Following application of the TRANSITION tool with an older patient in an acute medical ward, healthcare practitioner participants reflected that the tool was feasible and acceptable. Nurse participants reported that the tool was an acceptable and clear set of questions assisting them to screen and assess patients, engage patients in a conversation about their discharge, and support their understanding of the patient in the community. Most nurse participants commented that the tool supported them to know what to ask and assisted them to find more information about the patient at home as part of an informal conversation. They noted that this was not usual practice on their wards. One registered nurse (participant 1) noted ‘Being able to ask those questions that I do not necessarily ask. In terms of how well they are looking after themselves at home. Are they able to cook and clean for themselves? Because we don't always ask our patients that as nurses’.

Other nurse participants stated that the tool assisted them to continue the discharge planning initiated in the emergency department by the allied health professionals and to check for care needs that may have been missed. Allied health participants agreed with these comments and reported that they asked similar questions as relevant to their discipline in their own assessment tools. They reflected that the TRANSITION tool would be most useful for ward nurses to holistically assess patients. Most ward nurses agreed with these comments.

Evaluation findings also identified barriers to implementation of the communication tool in practice. All participants reported that the tool took between 10 and 25 min to use and some participants noted that the tool would be more feasible if more concise. Some ward‐based nurses considered that transitional care was not a part of their role. A registered nurse (participant 15) commented ‘From a nursing perspective, I guess I don't always have time or don't see my need to have to dig too deep into some of these questions [the TRANSITION tool] for the patient’. Other facilitators and barriers to the implementation of the tool as reported by evaluation participants are presented in Table 6.

**TABLE 6:** Evaluation: Facilitators and barriers to the implementation of the TRANSITION tool

Facilitators
Condense the tool and simplify questions Include the tool on the patient handover sheet Include the tool on a lanyard Include a question re how the patient is returning home? To be used by ward‐based nurses To be used by the nurse in charge Use as a checklist Use as a conversation guide Delegate a nurse to be responsible for completion of the tool Use the tool in the emergency department Use the tool on admission to the ward Convince practitioners that the tool is useful Include information re‐referral Align the tool with existing transitional care in the organisation to avoid replication of care processes Apply domains and questions as relevant to the patient and as relevant to the patient's readiness for discharge
Barriers
Perception of the tool as more paperwork Perception of some ward‐based nurses that transitional care is not their role Perception of some participants that only the patient's presenting issue should be the focus of discharge care

#### 3.3.2 Modification of the communication tool

We used the evaluation findings to modify the TRANSITION tool (presented in Table 5). The main modifications included adding a column labelled ‘date completed’ and making the tool more succinct to promote continuity and because ward‐based nurses had limited time to engage patients in conversations.

## 4 DISCUSSION

The study demonstrates how co‐design can be used to develop communication resources for implementation in the challenging area of transitional care. The TRANSITION tool is for use by ward‐based nurses to guide continuing conversations with patients, monitoring and referral to allied health services prior to the patient's discharge. As the only health professionals providing care over the 24‐h period, the TRANSITION tool can guide nurses in the continuing assessment of the relevance of the transitional care plan and any new or unidentified care needs. Findings across the three study phases indicate that engaging patients in transitional care and multidisciplinary communication strategies support transitional care. The TRANSITION tool can be used to strengthen these communication processes.

The current study findings are comparable with previous studies that have identified the importance of independence, quality relationships with healthcare practitioners, communication, and self‐care education for older patients during discharge from hospital to home (Bauer et al., 2009; Coleman et al., 2006; Hickman et al., 2015; Naylor et al., 2013). Findings also build on the existing knowledge base in communication and transitional care in multidisciplinary models.

Previous studies have examined communication at transitions as information exchange between healthcare practitioners (Foster & Manser, 2012; Jeffs et al., 2017). Evaluation findings indicate that through a discussion guided by the TRANSITION tool many nurse participants reported finding out information about the person at home thereby improving the fit of transitional care to the individual patient's care needs beyond their immediate inpatient stay. Nurse participants noted that identification of unmet care needs could prompt referral to allied health practitioners. Similarly, allied health practitioner participants commented on the main value of the communication tool for ward nurses to identify and refer issues to them for further support. Other research has also identified the effectiveness of multidisciplinary models of care where nurses and allied health practitioners work in teams to optimise transitional care for older patients living with chronic illnesses (Hickman et al., 2015). The current study is one example of how a communication tool can support multidisciplinary teamwork through ward nurses’ identification of care issues that they then refer to allied health practitioners.

The need for holistic transitional care planning from a multidisciplinary perspective that includes consideration of the patient at home is challenging for inpatient healthcare practitioners, patients and carers (Coleman et al., 2006). This is because the home environment and supports are generally unknown to inpatient practitioners and because patients’ recovery care needs may be unknown to patients and carers (Naylor et al., 2013). The use of the communication tool cannot be claimed to address these issues. However, findings indicate that in using the TRANSITION tool some ward nurses considered the patient's care needs at home and this was not their usual practice. This suggests that the communication tool may assist ward‐based nurses to improve person centred transitional care and bridge the hospital to home boundary.

In addition, the finding that some ward nurses’ use of the communication tool assisted them to learn more about the patient at home and that this was new to them indicates the importance of dialogue between older patients and ward nurses to identify patients’ real concerns. According to the nurse participants, older patients discussed their expectations about life at home after discharge and the hospital experience was less significant to them. Communication tools should guide nurses’ discovery of what matters to the patient to identify any unmet transitional care needs and optimise quality discharge and transitional care.

Safe quality transitional care is an expected quality standard in Australia and internationally including in relation to nursing in acute care settings. The view of some nurse participants in the evaluation phase that transitional care is not their role does not align with the expected quality and safety standards of multidisciplinary care inclusive of nurses in Australian healthcare (Australian Commission on Quality & Safety in Healthcare, 2019).

## 5 FURTHER RESEARCH

Further research is required to evaluate the acceptability and feasibility of the tool for patients and carers and to test the effectiveness of the communication tool across the patient transition into the community. The role of patients in using communication tools to take part in their own care transitions requires further investigation.

## 6 LIMITATIONS

There are several limitations to the current study. Conversational style communication involving questioning and discussion is a two‐way process. However, the current study only evaluated nurses and allied health practitioners’ use of the tool. The role of patients and of informal carers was not explored in the evaluation and their perspectives about using the communication tool are not known. We elected to sample healthcare practitioners from nursing, allied health and medicine across inpatient, rehabilitation and community settings and programs. Although this enabled a description of transitional care within a multidisciplinary model and across settings and programs, this did not permit a description of the important transitional care role of each individual health and social care discipline or of practitioners within a particular program or setting. No medical practitioners participated in the evaluation and their views are unknown. The study excluded patients living with cognitive difficulties. We are, therefore, unable to comment on the feasibility and acceptability of the communication tool with older patients with cognitive impairment during care transitions.

## 7 CONCLUSIONS AND IMPLICATIONS FOR PRACTICE

Despite these limitations, the current study describes how co‐design can enhance the involvement of service users alongside health practitioners in development of transitional care communication supports. There are also implications for nursing practice from this study. Findings across all study phases indicate that communication at ward level and prior to patient discharge that supports continuing planning, assessment and referral for older patients’ care transitions is an important focus for nurses within multidisciplinary models. Implementation of the TRANSITION tool into practice by ward‐based nurses can maximise the relevance of transitional care and complement care provided by allied healthcare practitioners. Evaluation findings further indicate that some nurses may perceive that transitional care is not their role in multidisciplinary models of care. Implementation of a communication tool such as the TRANSITION tool will require a carefully planned strategy to ensure an optimal fit with current practices and to engage ward‐based nurses. This will require education that includes explicit comparison between existing systems of care, practice gaps and how the communication tool could strengthen transitional care systems in healthcare services.

## CONFLICT OF INTEREST

The authors have no conflict of interest in this study.

## AUTHOR CONTRIBUTIONS

JA led the conceptualisation and design of all phases of the study with support from all co‐authors, JA undertook all data collection with support from two research assistants in the co‐design focus groups and evaluation phases, all authors contributed to data analysis, all authors contributed to the writing of the manuscript.

## Supporting information

Supplementary Material

Supplementary Material

## Data Availability

Data sharing not applicable.
