# Supplementary material for: Improving transitional care communication for older Australians from hospital to home: Co‐design of the TRANSITION tool
Source: Health Soc Care Community. 2022 May 4;30(6):e4223–38. doi: 10.1111/hsc.13816 (PMC10084314; doi:10.1111/hsc.13816)
Supplement: Supplementary file 1 — Supplementary Material [file HSC-30-e4223-s001.docx]

**Supplementary file 1**

Consolidated criteria for reporting qualitative studies (COREQ): 32-item checklist

| **No** | **Item** | **Guide questions/description** | **Page no in manuscript/comment** |
| --- | --- | --- | --- |
| **Domain 1: Research team and reflexivity** |  |  |  |
| Personal Characteristics |  |  |  |
| 1. | Interviewer/facilitator | Which author/s conducted the interview or focus group? | pp8-9  p11  p12  Table 2 |
| 2. | Credentials | What were the researcher's credentials? *E.g. PhD, MD* | p8  p13  Table 2 |
| 3. | Occupation | What was their occupation at the time of the study? | p8  p13 |
| 4. | Gender | Was the researcher male or female? | p8  p13 |
| 5. | Experience and training | What experience or training did the researcher have? | p8  p13 |
| Relationship with participants |  |  |  |
| 6. | Relationship established | Was a relationship established prior to study commencement? | No relationship was established. Noted as a footnote in Table 1. |
| 7. | Participant knowledge of the interviewer | What did the participants know about the researcher? e*.g. personal goals, reasons for doing the research* | Participants in each study phase were introduced to the research using the ethics approved Patient Information and Consent Form  This is noted under each phase in the methods section. |
| 8. | Interviewer characteristics | What characteristics were reported about the interviewer/facilitator? e.g. *Bias, assumptions, reasons and interests in the research topic* | None  Participants for each study phase were introduced to the research using the ethics approved Patient Information and Consent Form. |
| **Domain 2: study design** |  |  |  |
| Theoretical framework |  |  |  |
| 9. | Methodological orientation and Theory | What methodological orientation was stated to underpin the study? *e.g. grounded theory, discourse analysis, ethnography, phenomenology, content analysis* | Social constructivism |
| Participant selection |  |  |  |
| 10. | Sampling | How were participants selected? *e.g. purposive, convenience, consecutive, snowball* | Purposive sampling re each study phase. |
| 11. | Method of approach | How were participants approached? e*.g. face-to-face, telephone, mail, email* | Various methods of approach were used. These are explained under Participants and Procedure for each study phase. |
| 12. | Sample size | How many participants were in the study? | Table 2 |
| 13. | Non-participation | How many people refused to participate or dropped out? Reasons? | Nil. Not applicable |
| Setting |  |  |  |
| 14. | Setting of data collection | Where was the data collected? e*.g. home, clinic, workplace* | Reported under the methods section ‘setting’ and ‘procedure’ for each data collection method. |
| 15. | Presence of non-participants | Was anyone else present besides the participants and researchers? | Nil, not relevant. |
| 16. | Description of sample | What are the important characteristics of the sample? *e.g. demographic data, date* | Described in Table 2 |
| Data collection |  |  |  |
| 17. | Interview guide | Were questions, prompts, guides provided by the authors? Was it pilot tested? | Various data collection tools were developed and used. These are explained under ‘Data collection tools’ for each study phase and guided by systematic reviews of the literature. |
| 18. | Repeat interviews | Were repeat interviews carried out? If yes, how many? | No repeat interviews were carried out as this was not relevant to the study aims or rationale for each study phase. |
| 19. | Audio/visual recording | Did the research use audio or visual recording to collect the data? | Yes, noted on p6 |
| 20. | Field notes | Were field notes made during and/or after the interview or focus group? | Yes, noted on pages 11 and 14 |
| 21. | Duration | What was the duration of the interviews or focus group? | Table 2 |
| 22. | Data saturation | Was data saturation discussed? | Table 2 |
| 23. | Transcripts returned | Were transcripts returned to participants for comment and/or correction? | No member checks were carried out as this was not relevant to the study aims or rationale for each study phase. Participants in the co-design focus group considered the relevance and fit of interview findings from the context inquiry in relation to the study aim thereby supporting validation of interview data. |
| **Domain 3: analysis and findings**z |  |  |  |
| Data analysis |  |  |  |
| 24. | Number of data coders | How many data coders coded the data? | P13-14 |
| 25. | Description of the coding tree | Did authors provide a description of the coding tree? | Framework Approach was used. P13 |
| 26. | Derivation of themes | Were themes identified in advance or derived from the data? | Themes were derived from the data pp13-14 |
| 27. | Software | What software, if applicable, was used to manage the data? | Nil software was used. Not reported |
| 28. | Participant checking | Did participants provide feedback on the findings? | Participants in the co-design focus group considered the relevance and fit of interview findings from the context inquiry in relation to the study aim. |
| Reporting |  |  |  |
| 29. | Quotations presented | Were participant quotations presented to illustrate the themes / findings? Was each quotation identified? e*.g. participant number* | In findings  Table 3 |
| 30. | Data and findings consistent | Was there consistency between the data presented and the findings? | Yes, as per section re triangulation of findings and rigour (see Methods). |
| 31. | Clarity of major themes | Were major themes clearly presented in the findings? | pp14-18  Tables 3, 4, 5, 6 |
| 32. | Clarity of minor themes | Is there a description of diverse cases or discussion of minor themes? | pp14-18 |
