# Supplementary material for: Improving transitional care communication for older Australians from hospital to home: Co‐design of the TRANSITION tool
Source: Health Soc Care Community. 2022 May 4;30(6):e4223–38. doi: 10.1111/hsc.13816 (PMC10084314; doi:10.1111/hsc.13816)
Supplement: Supplementary file 2 — Supplementary Material [file HSC-30-e4223-s002.docx]

Supplementary file 2: Semi-structured interview guidelines

| **Study phase** | **Interview guidelines** |
| --- | --- |
| *Context inquiry* | |
| Semi-structured interviews (patients and informal carers) | 1. Tell me about your hospital discharge?   Prompts   1. What was the most important thing about your hospital discharge? 2. What do you value most about your hospital discharge? 3. What happened when you returned home? 4. What is the most valuable thing about coming home? 5. What services came to visit you at home? 6. What was most important about the support you received at home after discharge from hospital? 7. Were you satisfied with the way that the hospital discharged you? 8. Are you satisfied with the way that your community providers have supported you at home after your discharge? 9. What things were missing in your hospital discharge? |
| Semi-structured interviews (healthcare practitioners) | 1. Tell me what you know about the discharge of older people from hospital to home? 2. What is the best thing that has happened in the discharge of older people from hospital to home? 3. What is the most humorous thing that has happened in the discharge of older people from hospital to home? 4. What is the worst thing that has happened in the discharge of older people from hospital to home? |
| *Evaluation* | |
| Semi-structured interviews (healthcare practitioners) | 1. Tell me about using the TRANSITION tool?   Prompts:   1. What happened when you used the tool? 2. What was the most useful thing about using the tool? 3. What was the most valuable thing about using the tool? 4. Was the tool acceptable to you? 5. Was the tool feasible to use? 6. What was not useful about the tool? 7. What was not valuable about the tool? 8. What was not acceptable about the tool? 9. What was not feasible about the tool? 10. How could the TRANSITION tool be improved? |
